# Single-cell qPCR on dispersed primary pituitary cells -an optimized protocol

**DOI:** 10.1186/1471-2199-11-82

**Published:** 2010-11-12

**Authors:** Kjetil Hodne, Trude M Haug, Finn-Arne Weltzien

**Affiliations:** 1 Norwegian School of Veterinary Science, Department of Basic Sciences and Aquatic Medicine, PO Box 8146 Dep, 0033 Oslo, Norway; 2 University of Oslo, Department of Molecular Biosciences, PO Box 1041 Blindern, 0316 Oslo, Norway

## Abstract

**Background:**

The incidence of false positives is a potential problem in single-cell PCR experiments. This paper describes an optimized protocol for single-cell qPCR measurements in primary pituitary cell cultures following patch-clamp recordings. Two different cell harvesting methods were assessed using both the GH_4 _prolactin producing cell line from rat, and primary cell culture from fish pituitaries.

**Results:**

Harvesting whole cells followed by cell lysis and qPCR performed satisfactory on the GH_4 _cell line. However, harvesting of whole cells from primary pituitary cultures regularly produced false positives, probably due to RNA leakage from cells ruptured during the dispersion of the pituitary cells. To reduce RNA contamination affecting the results, we optimized the conditions by harvesting only the cytosol through a patch pipette, subsequent to electrophysiological experiments. Two important factors proved crucial for reliable harvesting. First, silanizing the patch pipette glass prevented foreign extracellular RNA from attaching to charged residues on the glass surface. Second, substituting the commonly used perforating antibiotic amphotericin B with β-escin allowed efficient cytosol harvest without loosing the giga seal. Importantly, the two harvesting protocols revealed no difference in RNA isolation efficiency.

**Conclusion:**

Depending on the cell type and preparation, validation of the harvesting technique is extremely important as contaminations may give false positives. Here we present an optimized protocol allowing secure harvesting of RNA from single cells in primary pituitary cell culture following perforated whole cell patch clamp experiments.

## Background

Using the patch clamp technique, we investigate the electrophysiological properties of single pituitary cells in culture [1], our main objective being to study the differences in electrical properties of follicle-stimulating hormone (FSH)- and luteinizing hormone (LH)-producing cells (gonadotropes) in teleost fish. Contrary to mammals, fish have separate cells producing FSH and LH, respectively [2-4]. Because a primary pituitary culture contains a mixture of endocrine cell types, it is imperative to discriminate between the cells investigated. Single-cell PCR may be used as a method to determine the identity of a cell in culture, and further to investigate its gene expression levels. Two harvesting techniques are commonly used in combination with single-cell PCR: harvesting of whole cells [5,6], and harvesting of cytosol only [7,8]. Harvesting of whole cells secures the entire cell content before lysis and PCR. However, there is a great risk of collecting extracellular solution during harvesting. Depending on cell culture type and conditions, the extracellular solution may contain RNA contaminations giving false results. To limit the risk of such contamination, cytosol may be harvested through a patch pipette using the tight giga seal whole cell patch clamp configuration. A drawback of cytosol harvesting is the potential loss of RNA if some cytosol is not harvested. Also, electrophysiological experiments are often dependent on using the perforated patch configuration, where the membrane patch is perforated by an agent added to the pipette solution, the antibiotic amphotericin B being one of the most commonly used agents [9]. This ensures electrical contact between the pipette solution and cytosol without diffusional loss of organic compounds from the cytosol. However, the progression from perforated patch to complete rupture of the membrane patch necessary for cytosol harvesting is often difficult to perform because the giga seal is easily lost in the process. Therefore, it may be required to compromise by performing electrophysiological experiments and gene expression analysis on different cells [10,11]. Fan and Palade [12] and Sarantopoulos *et al. *[13] successfully tested a more hydrophilic perforating agent, a saponin derived from the horse chestnut tree, called β-escin to reduce Ca^2+ ^current rundown in rat neurons. They concluded that β-escin also improves the giga seal formation and gives lower access resistance compared to traditional pore-forming antibiotics. In addition, Fan and Palade [12] reported that perforation of the membrane proceeds more rapidly using β-escin compared to nystatin, amphotericin B and gramicidin. Thus, β-escin could potentially improve the efficiency when harvesting cytosol for single-cell PCR. In this paper, we present a novel protocol for single-cell quantitative (q) PCR based on harvesting of cytosol through the patch pipette. The present study describes a protocol that reduces the risk of RNA contamination producing false positives, and at the same time facilitates the difficult transition from a perforated patch to a complete hole in the membrane necessary for cytosol harvesting.

## Methods

**Figure 1:**
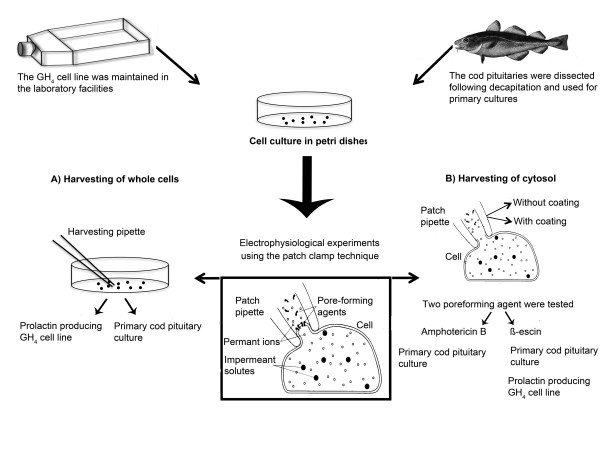
**Overview of the employed procedures and their validation for single-cell qPCR following electrophysiological recordings**. The present study involved two different cell culture systems. **Upper left**, the rat pituitary GH_4 _cell line producing prolactin was used initially to optimize cell harvesting. Later, the cell line was also used to compare harvesting efficiency. **Upper right**, the main objective of this study was to set up and validate a robust assay to identify follicle-stimulating hormone (FSH)-, and luteinizing hormone (LHβ)- producing cells based on their gene expression, using primary cultures of pituitary cells from maturing cod. The cell (**A**)- and cytosol (**B**)- harvesting techniques and protocols were also adjusted to be combined with electrophysiological experiments. **A) Harvesting of whole cells **was performed on both the GH_4 _cell line and the primary cell culture from cod pituitaries. Following cell harvest, the cell was transferred to a tube containing lysis solution and then reverse transcribed into cDNA. The cDNA was precipitated using ethanol before specific gene expression was amplified using a qPCR platform. **B) Harvesting of cytosol **was performed using the patch pipette following giga seal formation. The patch pipette was tested both with and without silanazing the glass surface. The method was evaluated on primary cell culture from cod pituitaries. Finally, two types of cell membrane perforating agents were evaluated on the primary culture from cod pituitaries, namely amphotericin B and β-escin. The combination of using silanized patch glass and β-escin as the perforating agent was evaluated on both the GH_4 _cell line and primary cell culture from cod pituitaries.

RNA molecules are relatively unstable and require particular consideration, especially before handling the small amount of transcripts found in a single cell. In order to prevent RNases from contaminating and degrading the RNA samples, we treated all equipment and experimental hardware with RNase-inactivating reagents, like RNaseZAP (Ambion, TX, USA). In addition, only aerosol resistant filter tips and certified RNase-free tubes and reagents were used. All glassware was baked overnight at 220°C, including glass capillaries used for making cell harvesting pipettes and patch electrodes. Figure 1 provides an overview of the methodological approach used in the present work.

### Cell cultures

#### GH_4 _cell line

The GH cell lines were originally derived from a rat pituitary tumor [14]. We have used the GH_4_C_1 _subclone, which predominantly secretes prolactin (PRL) [15]. Cells were grown in Ham's F-10 medium (Sigma, MO, USA) supplemented with NaHCO_3 _(14.3 mM), penicillin (100 U/ml), streptomycin (100 μg/ml; both from Invitrogen, CA, USA), horse serum (7.5%), and calf serum (2.5%; both from Med Probe, Oslo, Norway), pH 7.4. Cells were grown in 25 cm^2 ^tissue culture flasks and incubated at 37°C in a humidified atmosphere with 5% CO_2 _and 95% air. Once per week, the cultures were split by trypsin EDTA (200 μg/ml; Lonza, Belgium). Prior to the experiments, the cells were plated in 35 mm plastic petri dishes with a cell density of 20 000- 50 000 cells/cm^2^.

#### Cod pituitary primary cell culture

For making primary cell culture [16] we used maturing male and female Atlantic cod (1-3 kg) captured in the Oslo fjord and kept in the aquarium facilities at the University of Oslo. The holding tanks were continuously perfused with seawater with salinity of 28 ppt and a temperature of 7-8°C, and the fish were fed shrimps while in captivity. Immediately after decapitation, pituitaries (3-4 per culture) were transferred to ice-cold L-15 medium (Invitrogen) with penicillin (25 U/ml) and streptomycin (25 μg/ml; both from Invitrogen), 1.8 mM glucose, and pH adjusted to 7.8. Subsequently, pituitaries were washed in ice-cold phosphate-buffered saline (PBS; Invitrogen) with penicillin (25 U/ml) and streptomycin (25 μg/ml), at pH 7.8, chopped in approximately 1 mm^3 ^pieces with a scalpel and washed again. Tissue fragments were treated with trypsin (type II-S, 1 mg/ml; Sigma) in modified PBS for 45 min in a shaker water bath at 18°C. The tissue was incubated in the water bath for another 20 min with trypsin inhibitor (type I-S, 1 mg/ml; Sigma) in modified PBS. Tissue fragments were mechanically dissociated in ice-cold PBS using a plastic pipette. The cell suspension was filtered through a nylon mesh and centrifuged at 100 *g *at 4°C for 10 min before the cells were resuspended in growth medium (L-15 with 10 mM NaHCO_3_, 1.8 mM glucose, penicillin (25 U/ml), and streptomycin (25 μg/ml), pH 7.8). All solutions were adjusted to 320 mOsm using NaCl. The cells were plated out in 35 mm plastic petri dishes coated with poly-L-lysine (Sigma) with cell densities between 150 000 and 200 000 cells/cm^2^. The total yield from 3-4 pituitaries was 10-12 dishes. The dishes were kept at 12°C in a humidified atmosphere, with 0.5% CO_2 _and 99.5% air. The growth medium was changed after 24 hours to remove damaged and detached cells.

### Solutions

All chemicals were purchased from Sigma Aldrich and certified ultrapure or RNase-free. During the experiments, the growth medium was substituted with standard RNase-free extracellular solution (mM): 150 NaCl, 5 KCl, 2.4 CaCl_2_, 1.3 MgCl_2_, 10 (GH_4_C_1 _culture) or 1.8 (cod pituitary primary culture) glucose, 0.1% bovine serum albumin (BSA), 10 HEPES/NaOH, pH 7.4 (GH_4_C_1 _culture) or 7.8 (cod pituitary primary culture), adjusted to 320 mOsm. The patch pipette solution consisted of (mM): 120 CH_3_O_3_SK, 20 KCl, 20 sucrose, 10 HEPES/NaOH, pH 7.2, adjusted to 300 mOsm. The pipette solution for harvesting whole cells consisted of RNase-free extracellular solution where NaCl replaced BSA and glucose.

Two perforated patch methods were compared, using either amphotericin B (Sigma) [9] or β-escin (Sigma) [12] as perforating agents. Amphotericin B (600 μg/ml) was added to the pipette solution from a stock solution (60 mg/ml DMSO) and sonicated for 1 min. Fresh stock was prepared daily, pipette solution every hour. For β-escin, we used the procedure described by Sarantopoulos *et al. *[13]. In brief, β-escin (50 μM) was added to the pipette solution from a stock solution (25 mM in de-ionized nuclease-free water (Ambion)) and vortexed for 1 min. The stock was kept at -20°C for up to two weeks, the pipette solution prepared daily. The final pipette solution and stock was protected from direct light using aluminum foil.

### Harvesting techniques

Two harvesting techniques were compared in this work; harvesting of whole cells using a second harvesting pipette, and harvesting of cytosol using the patch pipette. Both techniques were applied following electrophysiological experiments using the patch clamp technique in the perforated patch configuration.

#### Harvesting of whole cells

Harvesting of single, whole cells was performed using a glass pipette with a tip diameter of 3-5 μm (1/3 to 1/2 of the cell diameter), made by a horizontal puller (P-97 Sutter Instrument CO, CA, USA). The pipette was filled with modified RNase-free extracellular solution, connected to an oil-filled cell-extractor (CellTram Oil, Eppendorf, Hamburg, Germany), and controlled by a hydraulic micromanipulator. During harvesting, the cells were observed using an inverted phase-contrast microscope. To avoid aspiration of possibly contaminated extracellular solution, the pipette was initially under positive pressure, created manually by regulating the cell-extractor. When the pipette was placed close to the cell to be harvested, the pressure was released and the cell trapped inside the pipette by applying moderate suction with the cell-extractor. The tip of the pipette containing the cell was passed several times through the air-fluid interface of the extracellular solution to remove potential debris adhering to the glass. The cell was then eluted into an RNase-free 1.5 ml tube (Ambion) with 10 μl lysis solution (Invitrogen) by applying a slight positive pressure. cDNA synthesis followed directly after harvesting, using the lysed cell solution as template.

#### Harvesting of cytosol using the patch pipette

As with the cell harvesting pipettes, the patch pipettes were made using the P-97 horizontal puller, with a tip diameter of approx 1 μm. The patch pipettes were silanized by briefly dipping the tip in Sigmacote (Sigma), a chlorinated organopolysiloxane, diluted in heptane, before being fire polished. The patch electrodes were filled with 3-4 μl of either amphotericin B- or β-escin-containing RNase-free intracellular solution. The pipette resistance was between 3.5 and 5.5 MΩ.

As the electrophysiological recordings were performed using perforated patch, a complete hole had to be made before harvesting of cytosol. A standard 20 ml syringe was used to generate the sub-atmospheric pressure needed to make the hole without breaking the giga seal. The syringe was also used for harvesting cytosol, by applying suction. In the initial experiments, the harvesting was terminated immediately after the giga seal broke. However, after the conditions had been optimized, harvesting was finalized only when the shrinkage of the cell halted, usually after about 3 minutes of harvesting. The pipette was then passed several times through the air-fluid interface of the extracellular solution before the content was transferred to an RNase-free 0.5 ml tube (Ambion) containing 7 μl of a sodium citrate EDTA- and EGTA- free solution (Ambion). This RNA storage solution also prevents potential inhibition of reverse transcription. To inactivate RNA-degrading RNases, we used 1 μl (40U/μl) RNasin Plus RNase Inhibitor (Promega, WI, USA). Positive pressure was applied while only the first few micrometers of the patch pipette was in contact with the solution in the tube, allowing the RNA situated in the first part to be ejected. While still maintaining positive pressure the patch pipette was then gently crushed against the bottom and the rest of the (RNA) solution in the patch pipette was emptied into the tube. For details see Sakmann and Neher [17].

### RNA isolation

In order to optimize the qPCR assay for identification of rat *PRL *mRNA in the GH_4 _cell line, 5000 cells per μl and a total of 10 000 cells were collected in 1.5 ml tubes and centrifuged at 10 000 *g *for 1 min, following rapid freezing in liquid nitrogen and stored at -80°C until further use. Cells were lysed in a total volume of 10 μl using CellsDirect's (Invitrogen) lysis enhancer (1 μl) and resuspension buffer (10 μl), before being incubated at 75°C for 10 min, and immediately placed on ice until cDNA synthesis. The same method was used also for lysis of single cells. cDNA synthesis was carried out directly after cell lysis.

To optimize the qPCR assays for cod *FSHβ*, *LHβ*, and elongation factor 1α (*EF1α*) RNA extracted from intact cod pituitaries were used. The pituitaries were collected immediately after decapitation and stored on RNAlater (Ambion) at -20°C until further processing. Two or three pituitaries were homogenized in 1 ml TRIZOL Reagent (Invitrogen), using a Fast Prep FP 120 machine (QBiogene, CA, USA). The extraction was performed using the manufactures protocol. The extracted total RNA was dissolved in water or RNA storage solution (Ambion) and stored at -80°C until cDNA synthesis.

The quality and quantity of the total RNA was evaluated using a NanoDrop spectrophotometer (NanoDrop Technologies, DE, USA). Only pure RNA with OD 260/280 between 1.8 and 2 was accepted. Except for RNA stored on RNA Storage Solution (Ambion), we also regularly checked that OD 260/230 was between 2 and 2.2.

### Reverse transcription (RT)

For first strand cDNA synthesis, 1 μl random hexamer primers (50 ng/μl) and 1 μl 10 mM dNTP mix (both from Invitrogen) were added to either the 10 μl of GH_4 _cell lysis solution from 10 000 cells and the single harvested cell solution, or to 3 μg of cod pituitary total RNA, or to the 11 μl of solution containing the harvested cytosol. The volumes were always adjusted to 13 μl using RNase free water (Ambion). The mixture was incubated for 5 min at 65°C, then 3 min incubation on ice. Subsequently, 4 μl 5X first strand buffer, 1 μl 0.1 M dithiothreitol (DTT), 1 μl RNase-out (40 U/μl), and 1 μl SuperScript III reverse transcriptase (200 U/μl; all from Invitrogen) were added, and the mixture was briefly centrifuged before incubation at room temperature for 5 min. The mixture was then incubated for 60 min at 50°C, before the enzyme reaction was inactivated by heating for 15 min at 70°C. The cDNA was stored at -20°C until qPCR. The same protocol was performed for both harvesting techniques, except that RNase-out was omitted for cytosol harvesting. After initial testing, the cDNA was also precipitated for removal of inhibitory factors directly following cDNA synthesis as described by Liss [18].

### Primer design

The qPCR primers where targeted to exon-exon borders, or alternatively, in the case of rat *PRL *where the intron was much longer than the amplicon, by placing the two primers on two different exons.

Except for the primers amplifying the whole coding region of cod *FSHβ *and *LHβ*, all primers were designed using the Primer3 software http://frodo.wi.mit.edu/primer3/input.htm. Potential primers were analyzed using the Oligo6 software (MedProbe, Oslo, Norway) to test for possible self-annealing and primer dimer formations (Table 1 for sequence details). The mRNA sequence from rat *PRL *(NM_012629) was retrieved from NCBI. Drs. Birgitta Norberg and Christian Mittelholzer at the Institute of Marine Research, Austevoll, Norway kindly provided cod *FSHβ *(DQ402373), *LHβ *(DQ402374) and *EF1α *(DQ402371) mRNA sequences. Contrary to the homogenous GH_4 _cell line, the primary culture from cod pituitary consists of 8 different hormone-producing cell types, meaning a significant number of cells harvested are not *FSHβ *or *LHβ*. Thus, in present study we used *EF1α *as a positive control ensuring positive detection for every cod pituitary cell or cytosol collected. Furthermore, as we found *EF1α *to be a more stabile expressed gene than *FSHβ *and *LHβ *it was also used during evaluation of the harvesting performance comparing the use of amphotericin B and β-escin. The primers were synthesized by MWG-Biotech AG (Ebersberg, Germany), diluted to 1 mM with nuclease free water (Ambion) upon arrival and stored at -20°C. From the stock solution, a working dilution of 5 μM was prepared.

**Table 1:** qPCR primers used in this study. Fw, forward; Rv, reverse.

Primer name		Primer sequence	Product length
Rat *PRL*	Fw	5'-CAT CAA TGA CTG CCC CAC TTC-3'	216 bp
		
	Rv	5'-AGC CGC TTG TTT TGT TCC TCA-3'	

Cod *FSHβ*	Fw	5'-GAA CCG AGT CCA TCA ACA CC-3'	63 bp
		
	Rv	5'-GGT CCA TCG GGT CCT CCT-3'	

Cod *LHβ*	Fw	5`-GTG GAG AAG AAG GGC TGT CC-3	81 bp
		
	Rv	5`-GGA CGG GTC CAT GGT G-3`	

Cod *EF1α*	Fw	5'-CCT TCA ACG CCC AGG TCA T-3'	100 bp
		
	Rv	5'-AAC TTG CAG GCG ATG TGA G-3'	

### Calibration curve for cod *FSHβ *and *LHβ *primer pairs

To assess the sensitivity of the primer pairs specific for cod *FSHβ *and *LHβ*, a qPCR calibration curve with defined numbers of molecules was made. Conventional PCR with primers defining the whole coding region was used [19], followed by gel electrophoresis. The PCR products were purified (Wizard PCR preps DNA purification system; Promega) and the concentrations were measured using a NanoDrop spectrophotometer. The number of molecules was calculated using the procedure described by Sambrook *et al. *[20]. Series of dilutions were made with 10- or 2- fold increments, ranging from 1-2 up to 10^5 ^molecules and subjected to qPCR analyses (see below). Detection limit was based on Cq values and on the quality of the melting curve for each qPCR reaction.

### qPCR

We developed and validated qPCR assays using SYBR green I detection dye on a LC480 platform (both from Roche, Basel, Switzerland). The final reaction volume of 10 μl contained 1 μl of 5 μM- forward and reverse primer, 5 μl of SYBR green I master mix and 3 μl of cDNA. In the non-template control (NTC), cDNA was substituted with 3 μl of nuclease free water. To evaluate possible genomic DNA interference, the primers for rat *PRL *were tested on 3 μl of undiluted cell lysate from 10 000 cells without the RT-step. For cod *FSHβ*, *LHβ*, and *EF1α*, we used 3 μl of 1 μM genomic DNA extracted with DNeasy Tissue kit (Qiagen, Hilden, Germany) from cod pituitary. All qPCR samples were run in duplicate, and for each plate we applied a NTC and positive control for each primer pair to control the experimental setup.

The qPCR reactions with primer pairs for rat *PRL *was carried out using an initial step of 10 min at 95°C to activate *Taq *polymerase, followed by 42-45 cycles consisting of 10 s at 95°C, 10 s at 59°C, and elongation at 72°C for 10 s. The fluorescence was measured at the end of each cycle. Crossing point (Cq) values were calculated using the "Second Derivative Maximum Method" [21]. To find the optimal qPCR conditions, a series of cDNA dilutions were made, and the Cq values were then plotted against the relative cDNA concentration. The qPCR efficiency was calculated using the slope of the regression line, according to the equation *E *= 10^[-1/slope]^. A slope of -3.32 gives *E *= 2, or a doubling of product for each PCR cycle. A melting curve analysis was performed directly following the PCR by continuously reading the fluorescence while slowly heating the reaction from 65°C to 98°C.

Due to higher melting temperature and shorter amplification sequences for the cod *FSHβ*-, *LHβ*- and *EF1α *primer pairs compared to the rat *PRL *primers, the annealing temperature was increased to 60°C and the elongation time was reduced to 5 s in the assays involving cod cDNA. The rest of the PCR protocol and melting curve analysis was the same as for rat *PRL*.

### Agarose gel-electrophoresis

To confirm amplicon size, qPCR products were analyzed by electrophoresis using a 1% agarose gel for rat *PRL *and a 2% agarose gel for cod *FSHβ*, *LHβ*, and *EF1α*. All gels were stained with 0.4 μg/ml EtBr. Before loading PCR products on the gel, 1 μl 10 × BlueJuice (Invitrogen) and 3 μl of nuclease free water (Ambion) were added to 2-3 μl of PCR product. Electrophoresis was conducted using an electrical field of 5 V/cm for 40-50 min.

### Sequencing

All qPCR products were regularly sequenced to assure specificity of the primers and to confirm that the different melting curve analyses were true positives. Unspecific, short nucleotide fragments and dNTPs were degraded from the qPCR reaction solution using exoSAP-IT (USB, OH, USA). Two μl qPCR solution, 2 μl of exoSAP-IT, and 3 μl water were mixed and incubated for 30 min at 37°C, and then inactivated for 15 min at 80°C. Forward primers (5 μM each) were added before the cDNA was sequenced at the sequencing platform (ABI 3730 high-throughput capillary electrophoresis sequencers) at the Department of Molecular Biosciences, University of Oslo.

### Statistical analysis

All statistics were calculated using GraphPad Prism version 4.0 or InStat version 3.0b.

The different experimental groups were first tested for equal variance followed either by unpaired t test with Welch's correction or for multiple group comparison Kruskal-Wallis test. Post test was performed using Dunn's Multiple Comparison Test. Numbers are given as mean ± SD if not otherwise stated. The levels of significance are P < 0.05* P < 0.01**, P < 0.001 *** and P > 0.05 not significant (ns).

## Results

**Figure 2:**
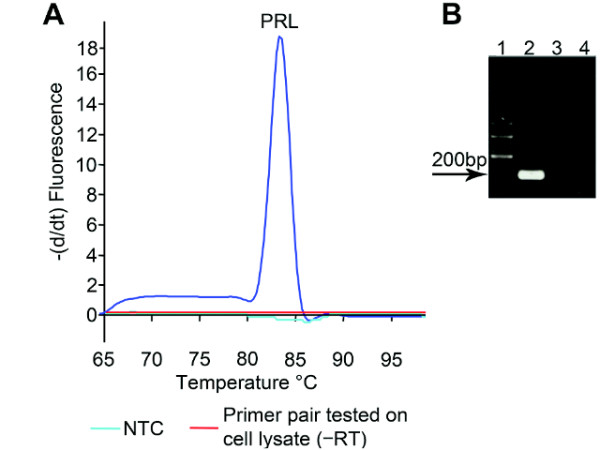
**Specificity of the SYBR green I assay for rat prolactin (*PRL*)**. **A**. Melting curve showing the negative change in fluorescence per time as a function of temperature. The specific peak in the melting curve indicates that the PCR has only amplified one product. The melting temperature (T_m_) for the PRL amplicon was 83.0°C. The non-template control (NTC), confirms the absence of primer dimers or other interfering factors of the assay besides the cDNA template. Substituting cDNA with cell lysate where the reverse transcription step was omitted (÷RT) showed no amplified product, confirming that genomic DNA did not interfere with the PCR results. **B **qPCR products visualized on a 1% agarose gel stained with EtBr. Lane **1**: 100 bp molecular ladder. Lane **2**: reverse transcribed cell lysate and specific primers for PRL amplifying one product of the expected size (216 bp). Lane **3**: specific primers for PRL tested on cell lysate without reverse transcription, confirming the primer pair's insensitivity for genomic DNA. Lane **4**: NTC for PRL.

**Figure 3:**
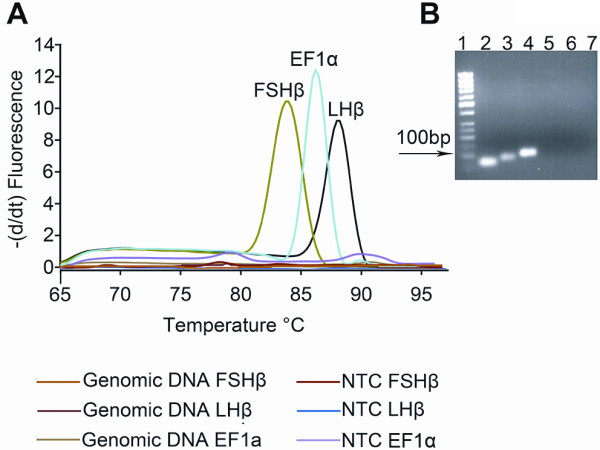
**Specificity of the SYBR green I assays for cod follicle-stimulating hormone beta subunit (*FSHβ*), luteinizing hormone beta subunit (*LHβ*), and elongation factor 1*α *(*EF1α*)**. Specific primers for cod *FSHβ*, *LHβ*, and *EF1α *mRNA were tested and validated. **A **Melting curve plotted as the negative change in fluorescence per time as a function of temperature. The specific melting peak for each qPCR product in the melting curve analysis indicates that only one product has been amplified. The specific melting temperature (T_m_) was for *FSHβ *84°C, *LHβ *88°C, and *EF1α *86°C. Non-template controls (NTC) performed by substituting cDNA with nuclease-free water as PCR template showed the absence of primer dimers or other interfering factors. Also, substituting cDNA with genomic DNA as PCR template showed no amplified product, confirming that genomic DNA did not interfere with the PCR results. **B **qPCR products visualized on a 2% agarose gel stained with EtBr. Lane **1**: 50 bp molecular ladder. Lane **2**: reverse transcribed pituitary RNA and specific primer pair for *FSHβ *showing one band with the expected size 63 bp. Lane **3**: reverse transcribed pituitary RNA and specific primer pair for *LHβ *showing one band with the expected size 81bp. Lane **4**: reverse transcribed pituitary RNA and specific primer pair for *EF1α *showing one band with the expected size 100 bp. Lane **6-8**: NTC for *FSHβ*, *LHβ*, and *EF1α*, respectively, confirming the absence of primer dimers and/or other interfering factors in the qPCR reaction.

The specificity of each qPCR primer pair was verified by first using a melting curve analysis followed by gel electrophoresis (Figure 2 for rat *PRL *and figure 3 for cod *FSHβ*, *LHβ*, and *EF1α*). In all samples tested using either cell extract (rat *PRL*) without the RT step or extracted genomic DNA (cod *FSHβ*, *LHβ*, and *EF1α*) showed no specific peaks, indicating absence of genomic DNA amplification (Figures 2a and 3a). Furthermore, all qPCR reactions were analyzed by agarose gel-electrophoresis (Figures 2b and 3b) and sequenced (data not shown) confirming that each primer pair were specific for cDNA only and that DNase treatment could be omitted.

**Figure 4:**
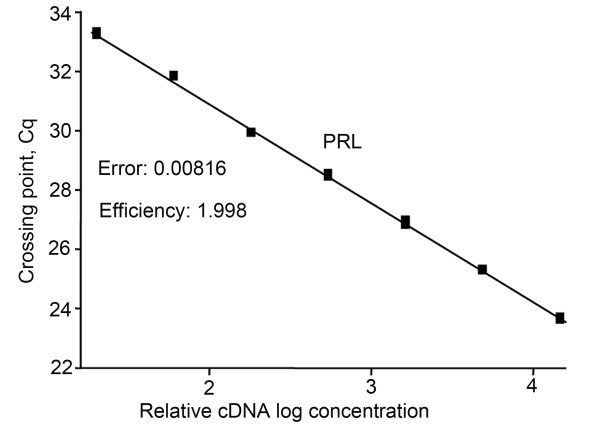
**Efficiency of the SYBR green I assay using specific primers for rat prolactin (*PRL*)**. **A**. Primer pairs that did not amplify genomic DNA were further tested using a dilution curve with three-fold cDNA (made from 10 000 GH_4 _cells) template dilutions between 1:20 and 1:14580. The crossing point (Cq; ordinate) values are plotted against the relative logarithmic concentration (abscissa) of the cDNA in the initial solution. The slope of the regression line is then used to calculate the efficiency of the qPCR assay. The assay for rat *PRL *had an efficiency of 1.998, equivalent to about 100%.

**Figure 5:**
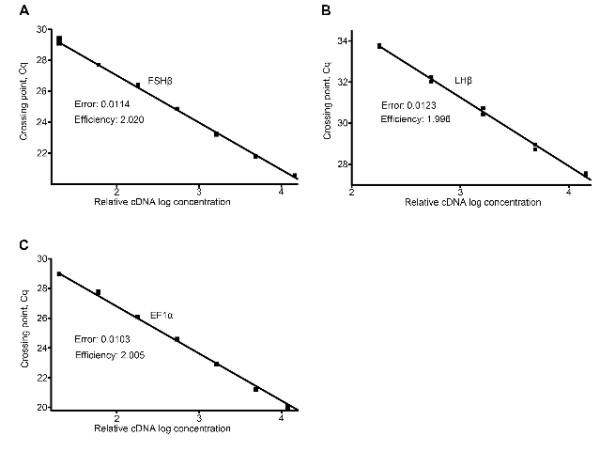
**Efficiency of SYBR green I assays using specific primers for cod follicle-stimulating hormone beta subunit (*FSHβ*), luteinizing hormone beta subunit (*LHβ*), and elongation factor 1*α *(*EF1α*)**. **A-C **A three-fold cDNA (made from 3 μg of cod pituitary total RNA) template dilution curve from 1:20 to 14580 (linearity down to 1620 times for LHβ) was made for each primer pair tested. The crossing point (Cq; ordinate) values are plotted against the relative logarithmic concentration (abscissa) of the cDNA in the initial solution. The slope of the regression line is then used to calculate the efficiency of the qPCR assay. The efficiency was 2.020 for the *FSHβ *primer pair, 1.996 for the *LHβ *primer pair, and 2.005 for the *EF1α *primer pair.

**Figure 6:**
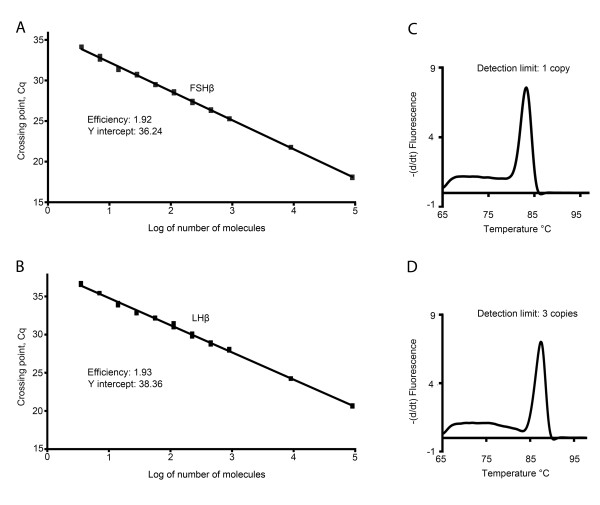
**Sensitivity of the follicle-stimulating hormone beta subunit (*FSHβ*)-, and luteinizing hormone beta subunit (*LHβ*) - primer pairs as measured in copy numbers**. By first amplifying the complete coding region of *FSHβ *and *LHβ*, we made a cDNA stock solution with defined molecule numbers. From the stock, a series of dilutions in triplicate with five- and two-fold increments was made starting from 100 000 molecules. Linearity for both the *FSHβ *(A) and *LHβ *(B) primer pairs were shown down to 3 molecules. The detection limit for the *FSHβ *primer pair was 1 copy (C), and for the *LHβ *primer pair it was 3 copies (D).

By using the slope of the regression line made from qPCR on a series of cDNA dilutions, the optimal conditions for the different primer pairs were adjusted. The optimized dilution curves in all assays showed efficiency close to 100% (Figures 4 and 5), meaning that for each PCR-cycle there is a doubling of the amount of amplicon. Additionally, because cod *FSHβ *and *LHβ *are the two main genes of interest in this study, we calculated the sensitivity of the FSHβ and LHβ primer pair based on the calibration curve (figure 6) with defined number of cDNA molecules. Linear relation down to 3 molecules was achieved for both *FSHβ *and *LHβ *(Figure 6a and 6b). The detection limit (mean ± SEM) for *FSHβ *(Figure 6c) is 1 molecule, Cq 35.91 ± 0.36 (n = 6), and for *LHβ *(Figure 6d) 3 molecules, Cq 36.05 ± 0.25 (n = 6).

In the following experiments we compared harvesting of whole cells using a harvesting pipette, with harvesting of cytosol using a patch pipette. We started with optimizing the conditions for harvesting whole cells on the GH_4 _cell line. For harvesting whole cells, the cell lysis solution CellsDirect contained all necessary compounds for exposing the RNA. Prior to cell harvesting, the Petri dishes containing the GH_4 _cells were thoroughly washed with extracellular solution. Control experiments where small amounts of extracellular solution were harvested confirmed the absence of RNA contamination that might affect the results.

The dilution curve resulting from using 10 000 GH_4 _cells (Figure 4) showed that 3 μl of cDNA diluted 1:20 gives a Cq value of about 24. Theoretically, an increase of 3 in the Cq value corresponds to approximately 10 × decrease in cDNA concentration, while an increase of 6 corresponds to a 100 × decrease in concentration. Hence, compared to the 1:20 dilution from 10 000 cells, 3 μl of undiluted cDNA from one cell would be expected to increase the Cq value by 7-8, to between 31 and 32.

**Figure 7:**
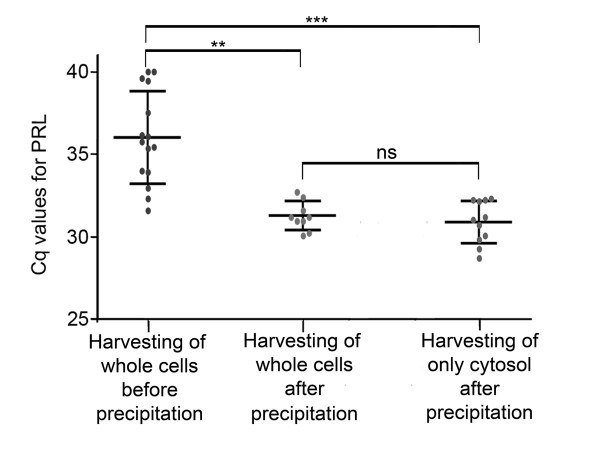
**Precipitation by ethanol of cDNA from single GH4 cells reduced the mean Cq value**. The grey dots in each chart representing individual Cq values for each cell- or cytosol-harvest with black lines representing mean ± SD. **First chart column**. Initially, qPCR on single cells was performed directly following reverse transcription, without any purification of cDNA. This gave a mean Cq value of 35.99 ± 2.82 (n = 15). **Second chart column**. After ethanol precipitation, the mean Cq value was reduced to 31.23 ± 0.88 (n = 9), achieving a significantly reduced mean Cq and significantly difference in variance (F-test with P = 0.0013). **Third chart column**. Harvesting of cytosol revealed no difference in mean Cq 30.87 ± 1.29 (n = 11) compared to harvesting of whole cells. The variance was similar (F-test with P = 0.14) when comparing the two harvesting techniques.

**Figure 8:**
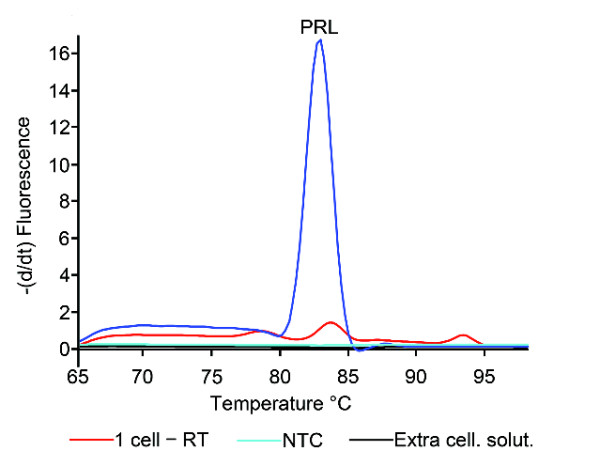
**Single-cell qPCR on rat (GH_4_) cells, using specific primer pair for rat prolactin (*PRL*)**. Directly following qPCR, the different samples were subjected to a melting curve analysis. The peak in the melting curve shows the presence of a rat *PRL*-specific amplicon in the sample containing a single cell. The melting temperature (T_m_) was 83.0°C, the same as for the amplified product from 10 000 cells. The lack of peaks in the melting curve analysis from the collected extracellular solution confirms the absence of amplified nucleotide and thus contaminating factors in the extracellular solution harvested together with the cell. The reverse transcription (RT) step was also routinely excluded from samples containing a single cell to confirm the primer pair's insensitivity to genomic DNA. For each qPCR run, a non-template control (NTC) was used substituting the cDNA with nuclease-free water in the qPCR mixture. The NTC melting curve analysis detected no primer dimers or contaminations in the reaction mixture.

Of the twenty GH_4 _cells harvested and analyzed for rat *PRL *by qPCR, seven had a Cq of 40 or above (Figure 7), while only two of these had a specific melting peak following melting curve analysis. The remaining 13 cells all showed a specific melting curve with a mean Cq of 35.99 ± 2.82 (n = 15). Five of these positive cells showed a small peak or a small elevation prior to the specific melting peak for rat *PRL *in the melting curve analysis, indicating suboptimal qPCR conditions or possibly presence of inhibitory factors in the solution. Previous studies [22,23] have shown that the enzyme reverse transcriptase constitutes a major inhibitory part of the PCR. This is even more critical when amplifying small amounts of cDNA, such as from single cells [18]. Thus, we precipitated by ethanol the cDNA synthesized from single cells prior to qPCR. After cDNA precipitation, single-cell qPCR reactions were performed as previously described. Following cDNA precipitation, we found only one out of the 10 cells tested to be negative for rat *PRL*, while the nine remaining cells were positive with highly specific melting curves. Precipitation of cDNA prior to qPCR also led to lower and more stable Cq values: 31.23 ± 0.88 (n = 9) (Figures 7 and 8 for melting curve analysis). This is a reduction in mean Cq of 4.7 ± 0.78, corresponding to a 25-27-fold difference in cDNA concentration. In addition, the variance (SEM) was significantly reduced following ethanol precipitation (P = 0.0026).

The technique for harvesting whole cells was subsequently evaluated on the primary cod pituitary culture. However, in addition to *EF1α*, mRNA for both *FSHβ *and *LHβ *were detected in all collected cells, contrary to the assumption that in fish, one cell synthesizes only one hormone. Control experiments by collecting 0.2-0.4 μl extracellular solution from multiple dishes, followed by qPCR, revealed extensive mRNA contamination in the bath with detection of all 3 genes.

The result of this extracellular contamination lead to a protocol for harvesting cytosol through the patch pipette. The tight seal made between the glass and cell membrane in connection with a patch clamp experiment is in the giga ohm range, leaving the inside of the patch pipette and its content unexposed to the possibly contaminated extracellular bath. For that reason, a stable giga seal is essential for both successful electrophysiological registration and for cytosol harvesting.

**Figure 9:**
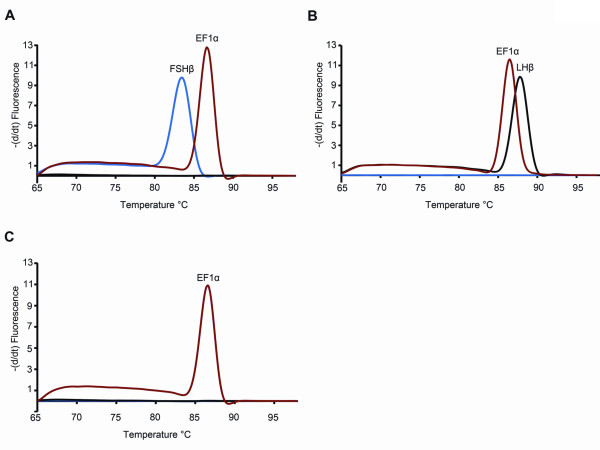
**Single-cell qPCR on primary culture from cod pituitary using specific primers for follicle-stimulating hormone beta subunit (*FSHβ*), luteinizing hormone beta subunit (*LHβ*), and elongation factor 1*α *(*EF1α*)**. Melting curve plotted as the negative change in fluorescence per time as a function of temperature. The different melting peaks for each qPCR product in the melting curve analysis indicates the specific phenotype of the cell. *EF1α *is only used as a positive control. Using a silanized patch glass, and β-escin as perforating agent prevented false positives and allowed reliable detection and to discriminate between FSH-, LH-, or non gonadotrope-producing cell. **A **cell positive for *FSHβ *and *EF1α *and negative for *LHβ *(black line without any specific peak). **B **cell positive for *LHβ *and *EF1α *and negative for *FSHβ *(blue line without any specific peak). **C **cell positive only for *EF1α *indicating neither a *FSHβ *(blue line) nor *LHβ *(black line) producing cell.

**Figure 10:**
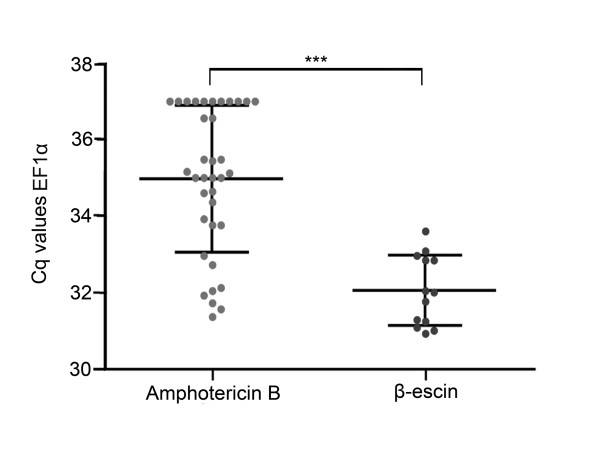
**Improved giga seal during harvesting of cytosol using β-escin as pore-forming agent**. The grey dots in each chart representing individual Cq values of the harvested cytosol from each cell with black lines representing mean ± SD. To evaluate and visualize the impact of the pore-forming agents amphotericin B and β-escin on preserving the giga seal during harvesting, we used *EF1α *as the target gene. The assay had an upper Cq detection limit of 37, and only samples where stable giga seal allowed cytosol to be collected for more than 10 s were analyzed. **First chart column**. Use of amphotericin B regularly lead to disruption of the giga seal soon after breaking the patch by suction, thus allowing harvesting for only a short period of time. The mean Cq value of *EF1α *using amphotericin B was 34.94 ± 1.9 (n = 36) with 11 of the samples reaching the upper Cq detection limit. **Second chart column**. Changing to β-escin significantly reduced the mean Cq to 32.04 ± 0.92 (n = 13) (p < 0.0001 ***), allowing most of the available RNA to be harvested. The mean difference in Cq was 2.91 ± 0.41 (mean ± SEM). The variance was also significantly different (F-test with P = 0.0051).

Although to a much lesser extent, mRNA contamination in the extracellular bath was still detectable after just leaving the patch pipette to rest close to the bottom of the petri dish. To prevent this contamination, we made the surface of the patch pipette hydrophobic by silanizing the glass using Sigmacote (Sigma). We found that diluting the Sigmacote 1:15 with heptane still excluded false positives but at the same time improved the condition for stable giga seal. Moreover, the hydrophobic pore forming antibiotic amphotericin B [1,9] also impedes the giga seal formation and maintenance. Often, the seal broke after a few seconds of cytosol harvesting making reproducible amounts of RNA for gene expression studies impossible. To facilitate and stabilize the conditions for cytosol harvesting, we therefore tested and evaluated the use of β-escin instead of amphotericin B. β-escin has to our knowledge not previously been used in cytosol harvesting experiments. Initial experiments on cod primary culture confirmed effective membrane perforation regularly giving access resistance < 15 MΩ. Compared to amphotericin B, β-escin also improved the stability during electrophysiological registrations. In addition, a stable giga seal could be maintained for more than 3 minutes. This enabled stable harvesting of cytosol, regularly observing cell shrinkage down to 1/3 or less of the initial size. The improved conditions allowed us to easily discriminate between *FSHβ *and *LHβ *producing cells and excluding possible false positives (Figure 9). To evaluate how well the giga seal was maintained during harvesting using either of the two pore forming agents, we compared the Cq values of the relatively stably expressing gene *EF1α *(Figure 10). Using amphotericin B as the perforating agent resulted in a mean Cq of 34.94 ± 1.9 (n = 36), while β-escin gave a mean Cq of 32.04 ± 0.92 (n = 13). This shows that harvesting was significantly improved by using β-escin. The observed difference in mean Cq of 2.9 ± 0.40 suggests, considering a *EF1α *assay efficiency of 2 or 100%, that about 6 to 10 times more RNA was harvested using β-escin. The variance was also significantly different between the two treatments (F test, P = 0.0051).

Finally, we also compared the two harvesting techniques, harvesting of whole cells and harvesting of cytosol, on the GH_4 _cell line (as this didn't produce false positives when harvesting whole cells), using rat *PRL *Cq values as an indicator of efficiency (Figure 7). Interestingly, no significant difference in Cq was detected between the two harvesting techniques. Mean Cq was 31.23 ± 0.88 (n = 9) for harvesting whole cells versus 30.87 ± 1.29 (n = 11) for cytosol harvesting, with mean difference of 0.35 ± 0.50. Also, there was no significant difference in variance between the two groups (F-test, P = 0.14).

## Discussion

The highly robust and sensitive method of qPCR has facilitated gene expression studies and has given new insights into single cell gene expression. The lognormal variation of copy number within single cells in a population [24] reflects the stochastic nature of how a cell continuously turns on and off its different genes [25-27]. In addition, increased demands for new sensitive and reliable techniques in clinical investigations using limited amount of sample [28-30] makes single-cell qPCR an attractive strategy. Therefore, it is essential to have robust and reliable protocols that discriminate between true negative and true positive results. The numerous protocols for securing single cells or cell content for gene expression studies provide possibilities for having different cell or tissue preparations [6-8,18,31-33]. The literature also thoroughly documents important optimization steps from cell lysis, to reverse transcription and qPCR [5,18,23,24,34-38].

However, we found that the literature provides rather limited information dealing with often unavoidable contaminations that may result in false positives [39]. A common solution to avoid false positives is to reduce the number of PCR cycles [39], but in our opinion, such a strategy may affect the amplification efficiency of the gene of interest. As such, it treats the symptoms rather than the root of the problem. Furthermore, the number of harvesting techniques suitable for securing the RNA is limited following electrophysiological experiments; harvesting of whole cells [23,24], or harvesting of only cytosol through the patch pipette [7,8]. Because perforating agents commonly used in patch clamp studies, often disrupt the giga seal during the necessary transition from a perforated membrane to a complete whole in the cell membrane, the latter method often excludes the possibility of combining perforated whole cell recordings with single-cell qPCR on the same cell.

In the present study, we have established a reliable method for gene expression analysis of single cells in primary culture following patch clamp experiments, focusing on *FSHβ*- and *LHβ*- producing cells from Atlantic cod. We evaluated the two harvesting strategies suitable in combination with the patch clamp technique; harvesting of whole cells and harvesting of cytosol through the patch pipette.

The novelty of our single-cell qPCR protocol is the combined use of the perforating agent β-escin and glass-pipette silanization allowing single-cell qPCR and perforated patch clamp experiments on the same cell. Together, this improves the harvesting efficiency and reduces the incidence of false positives.

The GH_4 _cell line is easily maintained and only needs gentle trypsination between cell passages. The cells also firmly attach to the dish surface even without coating. This is in contrast to the primary cell culture from fish pituitary, which needs both longer trypsination and mechanical dissociation for proper dispersal. Although we should expect variation in gene expression also in the homogenous GH_4 _cell line, this variation is less than what we find in a primary cell culture. The GH_4 _cell line is thus better for detecting and analyzing poor assay design and interassay variation. The initial work on the GH_4 _cell line proved valuable as it allowed analyzing and validating in detail the harvesting step and also the different reagents used. In addition, it allowed comparing the impact that different culture conditions have on the assay performance.

Following harvesting of the whole cell, the cell was transferred to a cell lysis solution to access the RNA. CellsDirect (Invitrogen) has been successfully used for reliable qPCR down to single cells in earlier studies [34,38], and was therefore chosen as a basis for cell lysis. Cell lysis and cDNA synthesis in the present study turned out to be readily transferable from 10 000 cells down to a single cell and no de-escalation of additives or additional steps was needed.

After cell lysis we reverse-transcribed the RNA to cDNA. The priming strategies of the reverse transcription between using oligo (dT), random primers, specific primers or combinations of these seem to be assay- and transcript-dependent [37,40-42]. Also, a newly developed method for single-cell gene expression analysis uses oligo (dT) immobilized beads to obtain mRNA [38]. However, one important drawback of using oligo (dT) is that mRNA with degraded poly(A) tail will not be reverse transcribed. Instead, random primers can potentially bind to all regions of an RNA fragment increasing the chance of converting RNA fragments complimentary to the designed PCR primers. Interestingly, combining random primers and oligo (dT) have been successfully used for single-cell analysis [5,37]. In future studies the use of both random primers and oligo (dT) will be further evaluated, especially for low abundant transcripts like receptors and ion channels.

As with priming strategies, great variation of the enzyme performance of the reverse transcriptase on different genes is observed [36]. Therefore, for quantitative measurements the same enzyme should be used between every sample. In our laboratory we have tested different types of reverse transcription enzymes and support earlier results [38] that SuperScript III reverse transcriptase gives high efficiency even when having low transcript levels.

All primers were tested and confirmed insensitive towards genomic DNA. We could therefore omit the extra step of DNase treatment. The primers were further evaluated using a cDNA dilution curve. For *FSHβ *and *LHβ*, it proved crucial to have relatively short amplicons for an optimal assay with high specificity and efficiency, but also it allowed us to reduce the elongation time of the PCR thereby reducing chances of amplifying genomic DNA [34]. We also observed that small variations in primer annealing temperature greatly affected the qPCR assay efficiency. In general, increased PCR efficiency and sensitivity are obtained by reducing the primer annealing temperature. However, too low annealing temperature can potentially lead to unspecific binding of primers and create unspecific or multiple peaks in the melting curve, and thus detection of false positives.

Several factors in the reverse transcription mixture, including reverse transcriptase, inflict with the PCR and inhibit the polymerase enzyme [22,23]. To overcome the inhibitory effects, two strategies have been tested and validated down to single cells: either to use as low concentration of the reverse transcription agents as possible [5], or to remove the inhibiting reagents using ethanol precipitation adapted to low transcript levels [18]. The present study substantiates the importance of ethanol precipitation for reliable gene detection in single cells, significantly stabilizing and reducing the mean Cq values (Figure 7). Furthermore, ethanol precipitation of cDNA may help to remove any distorting salt compounds found in the RNA storage solution and pipette solution.

During the optimization of the different harvesting strategies, we revealed some interesting differences between the initial GH_4 _cell line test model and the primary dispersed cell culture from fish pituitary. The main difference being the prolonged trypsination and mechanical dissociation needed to disperse the cells from fish pituitaries. This treatment thus leads to increased contamination of RNA in the bath. In addition, we had to minimize washing of the Petri dishes with extracellular solution because the primary pituitary cells detached, making it impossible to conduct electrophysiological experiments. Because some extracellular fluid inevitably is collected together with the harvested cell, we had to exclude harvesting of whole cells as a method for obtaining RNA.

Surprisingly, we still experienced false positives after changing to cytosol harvesting through the patch pipette. The most probable cause is the ability RNA has to attach to charged residues on the glass surface. Based on these assumptions we therefore silanized the glass using Sigmacote. This proved to be crucial for avoiding possible interference from unwanted RNA. Moreover, we could dilute the Sigmacote still excluding RNA from attaching, but significantly improving the giga seal formation. The improved seal frequency and tightness after diluting Sigmacote has also been described by Przysiezniak and Spencer [43] using neurons from a hydrozoan jellyfish.

One very important implication concerning cytosol harvesting through the patch pipette is the difficulties of maintaining a stable giga seal during harvesting. In most cases we experienced seal breakage after a few tenths of a second into the harvesting when using amphotericin B as the perforating agent. To overcome this limitation we tested a fairly new agent in the context of electrophysiology [12,13] named β-escin. This agent has been validated for use as a pore forming agent in patch clamp experiments but never for cytosol harvesting. In agreement with earlier studies [12,13] we found β-escin to easily perforate the cell membrane giving low access resistance (< 15 MΩ). Furthermore, β-escin stabilized the giga seal facilitating the difficult transition from a perforated patch to a complete hole in the membrane followed by harvesting of cytosol. Using *EF1α*, a more stably expressed gene compared to *FSHβ *and *LHβ *allowed us to evaluate harvesting efficiency (Figure 10). The significant difference in variance between using amphotericin B and β-escin we believe reflects the unstable giga seal using amphotericin B. This instability in most cases leads to a reduced harvesting because the harvesting procedure has to be aborted as soon as the giga seal breaks to prevent extracellular contamination.

For quantitative gene expression studies using single cells it is of course plausible to favor harvesting of the whole cell, as this in most cases assures that all the RNA is collected. However, when comparing the two harvesting methods for obtaining RNA on the GH_4 _cell line, we found no significant difference in Cq values (Figure 7). This shows that equal amounts of RNA were obtained when harvesting only cytosol compared to harvesting the whole cell.

## Conclusion

In conclusion, we have developed a single cell qPCR assay for reliable detection of *FSHβ *and *LHβ *from primary dispersed fish pituitary cells. The use of silanized patch pipettes for cytosol harvesting reduces the risk of contaminating the samples with RNA from the extracellular solution. In addition, using β-escin as the perforating agent in perforated patch clamp experiments allows us to progress from a perforated patch to the traditional whole cell configuration followed by cytosol harvesting without losing the giga seal, which is necessary for reliable harvesting.

## Authors' contributions

KH carried out the analyses under supervision of TMH (cell culture, cell/cytosol harvesting) and FAW (molecular biology), participated in the design of the study, and drafted the manuscript. TMH and FAW conceived, designed, and financed the study, and participated in the writing process. All authors read and approved the final manuscript.
